# HIV viral suppression among pregnant and breastfeeding women in routine care in the Kinshasa province: a baseline evaluation of participants in CQI‐PMTCT study

**DOI:** 10.1002/jia2.25376

**Published:** 2019-09-08

**Authors:** Marcel Yotebieng, Christian Mpody, Noro LR Ravelomanana, Martine Tabala, Fathy Malongo, Bienvenu Kawende, Paul Ntangu, Frieda Behets, Emile Okitolonda, for the CQI‐PMTCT study team

**Affiliations:** ^1^ Division of Epidemiology College of Public Health The Ohio State University Columbus OH USA; ^2^ School of Public Health The University of Kinshasa Kinshasa Democratic Republic of Congo; ^3^ National AIDS Control Program (PNLS) Provincial Coordination Kinshasa Democratic Republic of Congo; ^4^ Department of Epidemiology Gillings School of Global Public Health The University of North Carolina at Chapel Hill Chapel Hill NC USA; ^5^ Department of Social Medicine The University of North Carolina at Chapel Hill Chapel Hill NC USA

**Keywords:** pregnant women, option B+, treat all, universal coverage, viral suppression, vertical transmission, viral load monitoring, quality of care, HIV

## Abstract

**Introduction:**

Published data on viral suppression among pregnant and breastfeeding women in routine care settings are scarce. Here, we report provincial estimates of undetectable and suppressed viral load among pregnant or breastfeeding women in HIV care in Kinshasa, Democratic Republic of Congo (DRC) and associated risk factors.

**Methods:**

This cross‐sectional study was conducted as part of a baseline assessment for the CQI‐PMTCT study: an ongoing cluster randomized trial to evaluate the effect of continuous quality interventions (CQI) on long‐term ART outcomes among pregnant and breastfeeding women (NCT03048669). From November 2016 to June 2018, in each of the 35 Kinshasa provincial health zones (HZ), study teams visited the three busiest maternal and child health clinics, enrolled all HIV‐positive pregnant or breastfeeding women (≤1 year post‐delivery) receiving ART, and performed viral load testing. Log binomial models with generalized estimating equations to account for clustering at the HZ level, were used to estimate prevalence ratios comparing participants with undetected (<40 copies/mL) or suppressed (<1000 copies/mL) viral load across levels of individual and site characteristics.

**Results:**

Of the 1752 eligible women, 1623 had viral load results available, including 38% who had been on ART for <6 months and 74% were on tenofovir‐lamivudine‐efavirenz. Viral load was undetectable in 53% of women and suppressed in 62%. Among women who were on ART for ≥12 months, only 60% and 67% respectively, had undetectable or suppressed viral load. Viral load was undetectable in 53%, 48% and 58% of women testing during pregnancy, at delivery, and in postpartum respectively. In multivariable log binomial models, duration of ART >12 months, older age, being married, disclosure of HIV status, receiving care in an urban health zone or one supported by PEPFAR were all positively associated with viral suppression.

**Conclusions:**

The observed high level of detectable viral load suggests that high ART coverage alone without substantial efforts to improve the quality of care for pregnant and breastfeeding women, will not be enough to achieve the goal of virtual elimination of vertical HIV transmission in high‐burden and limited resources settings like DRC.

## 1 Introduction

Spurred by the Global Plan towards the elimination of new HIV infections among children by 2015 and keeping their mothers alive, new paediatric HIV infections were reduced by 60%, between 2009 and 2015, in the 21 countries in sub‐Saharan Africa with the highest burden 1. Galvanized by such progress, the Joint United Nations Programme on HIV/AIDS (UNAIDS) and the U.S. President's Emergency Plan for AIDS Relief (PEPFAR), launched in 2016 the *Start Free, Stay Free, AIDS Free* initiative with super‐fast‐track target of reducing the number of newly infected children to less than 40 000 by 2018 and 20 000 by 2020 2. To achieve this ambitious target, 95% of pregnant women living with HIV (PWLHIV) must be reached and sustained on lifelong antiretroviral therapy (ART) in 21 sub‐Saharan African countries which together with India and Indonesia, account for about 90% of new paediatric infections 2.

Although UNAIDS monitors and regularly publishes updated ART coverage and estimates of viral suppression 3, population based studies on viral suppression (i.e. third 90) among PWLHIV are limited 4. Yet, in addition to the high rate of loss to follow‐up among PWLHIV 5, adherence to ART among those retained in care is known to be sub‐optimal, particularly in the postpartum period 6, 7. In a pooled analysis of 51 studies involving 20,153 PWLHIV mostly from United States, Kenya, South Africa and Zambia, 75.7% of participants had adequate (≥80%) ART adherence during pregnancy compared with 53.0% in the postpartum 8. Similarly, in an analysis of data from women who initiated ART during pregnancy and breastfeeding from 13 large health facilities in Malawi between September 2011 and October 2013, about 70% were found to have adequate (>90% of days covered by pharmacy claims) adherence during the first two years of ART. However, only about 30% of them maintained adequate adherence at every visit 9. A recent report from South Africa showed that, even after viral suppression had been achieved, only about 70% of women were able to maintain viral suppression throughout a median follow‐up time of 322 days 10. Because routine monitoring of viral load is limited in most of the 21 priority countries in sub‐Saharan Africa, to the best of our knowledge there has been no attempt outside of targeted cohort studies, to estimate the proportion of pregnant and breastfeeding women with viral suppression in routine ART care.

The aims of this study were to 1) estimate the proportion of pregnant and breastfeeding women receiving routine HIV care in maternal and child health (MCH) clinics in the Kinshasa province of the Democratic Republic of Congo (DRC) with undetectable (<40 copies/mL) or suppressed (<1000 copies/mL) viral load and 2) assess socio‐demographic, clinical and health facility characteristics associated with undetectable and suppressed viral load.

## 2 Methods

### 2.1 Study design and settings

This cross‐sectional study was conducted as part of baseline assessment for the CQI‐PMTCT study: an ongoing cluster randomized trial to evaluate the effect of continuous quality interventions (CQI) on long‐term outcome of ART among pregnant and breastfeeding women (NCT03048669) 11. Briefly, Health zones were randomized to data driven quality improvement group in which multidisciplinary teams are brought together quarterly to identify key bottlenecks in care delivery system, develop and implement plans to address the bottlenecks at the district or facility levels. The protocol with detailed description of the intervention has been published elsewhere 11.

DRC is one of the *Start Free, Stay Free, AIDS Free* priority countries 2. Roughly nine in ten pregnant women in DRC attend at least one antenatal care (ANC) visit, surpassing 97% in Kinshasa 12. Starting in October 2013 and with support from PEPFAR and the Global Fund to Fight AIDS, Tuberculosis and Malaria, DRC has been progressively scaling‐up Option B+ (ART for life to all HIV‐positive pregnant women) 13. In Kinshasa, all pregnant women with unknown HIV status registering for antenatal care or presenting for delivery are offered an HIV test (acceptance rate > 97%) and those who test positive are immediately initiated on a triple antiretroviral combination. After delivery, mother‐infant pairs are followed up at MCH facilities until 24 months or the cessation of breastfeeding or until the mother (and the child if HIV positive) is transferred to HIV clinics for continued HIV care. Viral load measurement is accessible at the national reference laboratory in downtown Kinshasa at a subsidized cost of $25 or at higher cost per test at two other facilities and is recommended for every patient on ART at least once a year.

### 2.2 Study population and data collection

**Figure 1:**
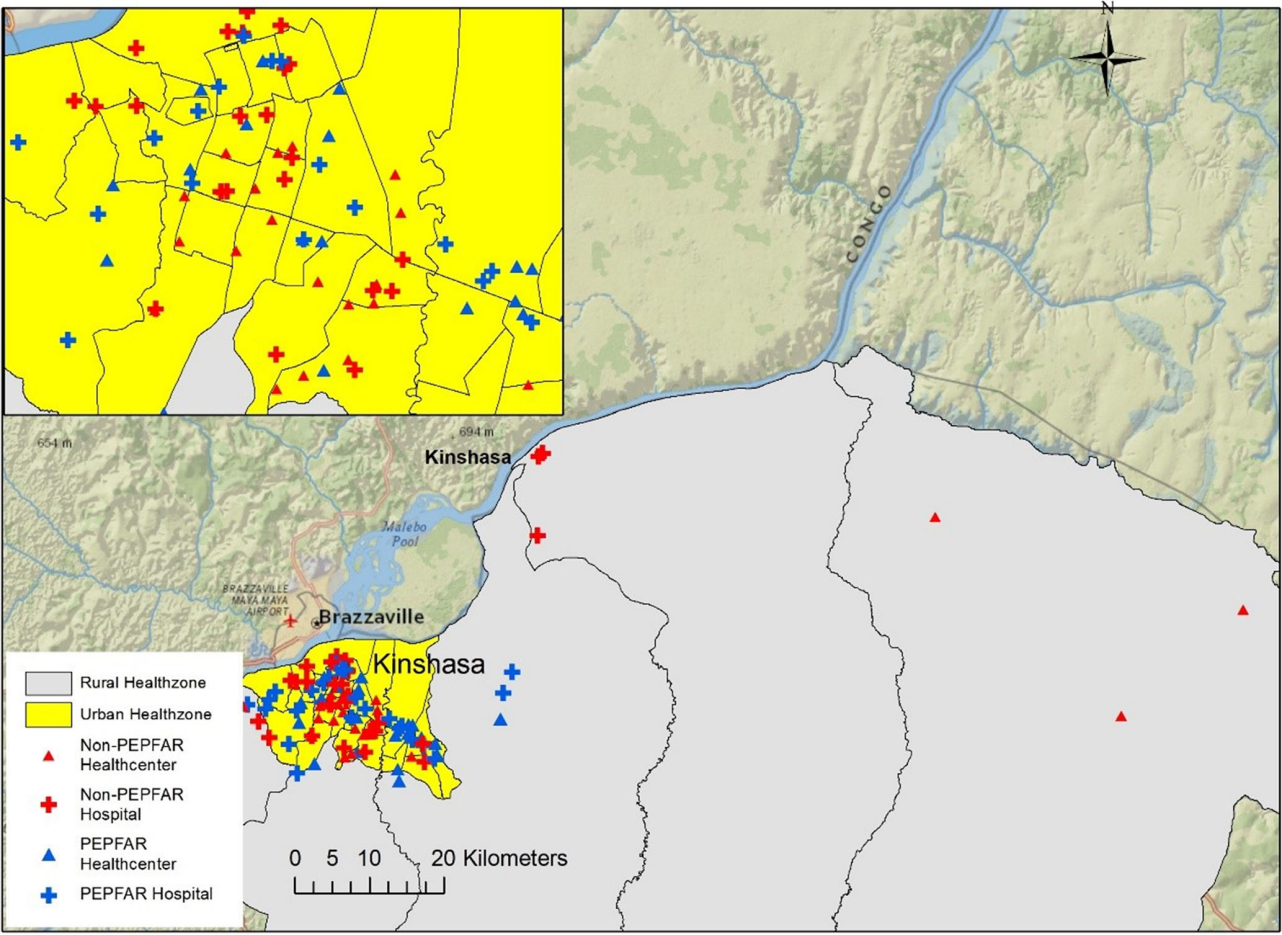
Map and characteristics of enrolment sites.

From a list of MCH facilities providing services to prevent mother‐to‐child transmission of HIV (PMTCT) obtained from the National Program for the Fight against AIDS (PNLS), we selected the busiest three in terms of number of PWLHV served during 2015 in each of the 35 health zones of the Kinshasa province (Figure 1).

Between June and November 2016, study staff visited each selected health facility to collect information on the facility characteristics using a structured questionnaire. Following the facility survey, starting November 2016, all pregnant or breastfeeding women living with HIV receiving care in selected facilities were identified using medical records, approached during routine clinic visits and invited to participate in the study. Participants could be enrolled at any time during pregnancy, immediately after delivery (in the maternity ward during the one to three days post‐delivery observation), or during well‐child visits in postpartum period. Eligible women who were not on ART, no longer breastfeeding, or whose infants were more than 12 months old were excluded. After obtaining written informed consent, a structured questionnaire was used to collect participant's socio‐demographic and clinical information.

### 2.3 Viral load testing

For participants who agreed to provide a blood specimen, five spots of 50 mL of blood were obtained via a finger prick on Whatman paper. The collected blood spots were then dried at ambient temperature for at least three hours. The dried blood samples (DBS) were then packaged with desiccant and stored at −20°C until testing at the National AIDS Reference Laboratory in Kinshasa (LNRS). LNRS proceeds samples on the basis of first come, first served, meaning it may take weeks or sometime months for results to become available. The median time between sample collection and availability of results was 72 days. Viral load testing was conducted using m2000rt Real‐Time HIV‐1 assay (Abbott, Chicago, IL), with a detection limit of 40 copies/mL. The LNRS laboratory received technical support including external quality assurance 14.

### 2.4 Variables

The primary outcomes in this study were viral suppression defined as viral load <1000 copies/mL and undetectable viral load or viral load <40 copies/mL. Other variables of interest included facility and participant demographic and clinical characteristics. Facility characteristics included: location (urban vs. peri‐urban/rural), PEPFAR funding support for HIV care (Yes vs. No), type (hospital vs. health centre). Participant demographic and clinical characteristics included: maternal age in years (≤ 24, 25 to 34, or 35+), marital status (married/ cohabitating vs. divorced/separated/widowed/never married), timing of HIV diagnosis (prior to current pregnancy vs. during current pregnancy), duration of treatment in months (<6, 6 to 12, 12 to 24, or 24+), educational level (primary, secondary or tertiary), usual mode of commute to the clinic (walking vs. other), disclosure of HIV status to anyone (yes vs. no), primigravida (no vs. yes), timing of viral load testing (pregnancy, immediately after delivery, or post‐partum) and a wealth index score used as a proxy of socioeconomic status (SES). The wealth index score was obtained, as in previous manuscripts 15, 16, from principal components analysis of the following factors: years of education (in years), average number of household members per room, number of sleeping beds in the household, type of household water source (communal vs. private pipe), cooking fuel type (electrical stove vs. wood/charcoal) and ownership status (yes vs. no) for several household's goods (mobile phone, radio, fridge, vehicle, bike and motorcycle). The first component explained 20.7% of variability in the dataset and was categorized into quintiles: 0 (lowest SES), 1, 2, 3 and 4 (highest SES). The duration of ART was calculated using the date of ART initiation extracted from participants’ medical records. When the date was not available in clinic records, participants were asked to estimate about how long they have been on ART.

### 2.5 Statistical analysis

The proportion of women with viral suppression (viral load <1000 copies/mL) 13 and undetectable viral load were estimated by time of enrolment: during pregnancy, immediately after delivery and postpartum. Bivariable and multivariable log binomial models were used to estimate the prevalence ratio (PR) and 95% confidence interval (95% CI) as measure of the strength of the association between health facility, sociodemographic and clinical characteristics with viral suppression. When the log binomial did not converge, Poisson regression modelling was used. Generalized estimating equation was used in all models to account for potential clustering at the level of health zone or possible extra Poisson variance. Only facility and individual characteristics found to be statistically associated with viral suppression in bivariable analysis (alpha = 0.2) were included in multivariable models 17. All statistical analyses were conducted using Stata Version 14.0 and all statistical tests were two‐sided with an alpha level of 0.05 except when otherwise indicated.

The study was approved by the University of Kinshasa School of Public Health Ethical Review Committee and the Ohio State University Institutional Review Board. All participants provided written inform consent.

## 3 Results

### 3.1 Participants characteristics

**Figure 2:**
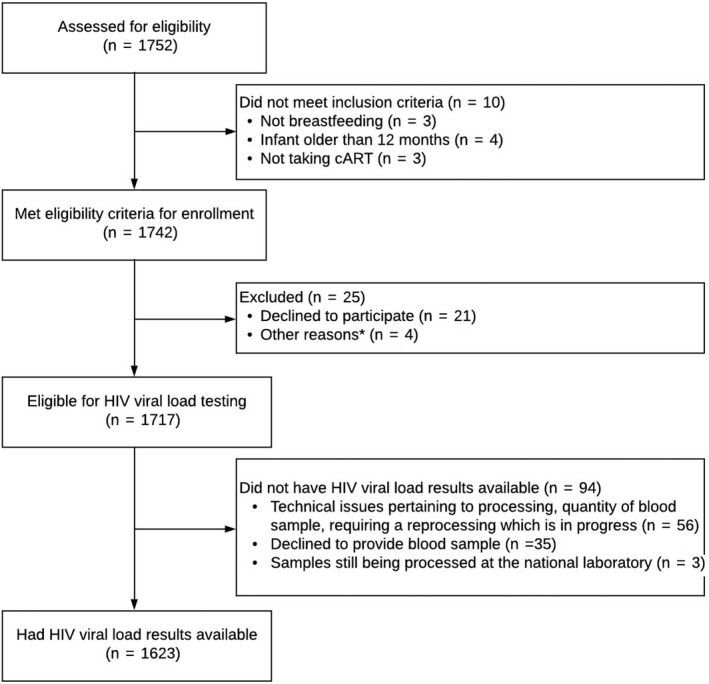
Participants recruitment flowchart. *Other reasons include hearing impairment of participant, intent of participant to transfer to a different clinic, false pregnancy.

By 30 June 2018, when the database was closed for this analysis, 1752 potential participants had been assessed for participation in the study, of whom 1742 met eligibility criteria and 1717 were enrolled. Viral load results were available for 1623 women (Figure 2).

Table 1 summarizes the distribution of facility and individual characteristics of 1623 participants included in this analysis. Over half (56%, n = 914) of women were enrolled in hospitals as opposed to health centres; 62% (n = 1012) in health facilities supported by PEPFAR; and 93% (n = 1510) in facilities located in urban areas.

**Table 1:** Socio‐demographic and clinical characteristics of 1623 women living with HIV from 105 clinics in Kinshasa tested for HIV viral load between November 2016 and July 2018^a^

Characteristics	No %^b^
Timing of viral load testing	
Pregnancy	873 (54)
Delivery	378 (23)
Post‐partum	378 (23)
Location of health facility (where participant was recruited)	
Peri‐urban/rural	113 (7)
Urban	1510 (93)
PEPFAR funding of facility	
No	611 (38)
Yes	1012 (62)
Type of facility	
Health centre	709 (44)
Hospital	914 (56)
Time on ART (median [IQR])	17 [2, 46]
<6 months	602 (38)
6 to 11 months	119 (8)
12 to 24 months	176 (11)
>24 months	679 (43)
Marital status	
Divorced/separated/ widowed/never married	511 (32)
Married/cohabitating	1078 (68)
Maternal age in years (median [IQR])	32 [27, 36]
≤24	239 (15)
25 to 34	848 (53)
35+	502 (32)
Disclosure of HIV status^c^	
No	787 (49)
Yes	826 (51)
ART regimen	
TDF + 3TC+FEV	1208 (74)
AZT + 3TC+NVP	207 (13)
Other	208 (13)
Mode of transport to the clinic	
Walking	601 (38)
Taxi/other	988 (62)
Primigravida	
Yes	145 (9)
No	1445 (91)
Educational level	
Primary	221 (14)
Secondary	1118 (70)
Tertiary	249 (16)
SES in quintile^d^	
0 (Lowest)	295 (21)
1	274 (19)
2	289 (20)
3	271 (19)
4 (Highest)	297 (21)

ART, antiretroviral therapy; SES, socio‐economic status.

^a^The analytical sample was derived from the enrolment data of an ongoing cluster randomized controlled trial, aimed at evaluating the effect of data‐driven continuous quality improvement on long‐term ART outcomes in Kinshasa, Democratic Republic of Congo; ^b^frequencies might not add up to the total for the category, because of missing data; ^c^self‐report of disclosure of HIV status to anyone; ^d^calculated using principal component analysis and categorized in five quintile groups.

The median age of participants was 32 (Interquartile range (IQR) 27 to 36) with 15% (n = 239) of women aged 24 years or younger and 53% (n = 848) aged 25 to 34 years. Most participants (68%, n = 1078) were married or cohabiting, 14% (n = 211) had completed less than primary school and 16% (n = 249) had at least some tertiary education; and 38% (n = 601) reported walking to their clinic.

Overall, 54% (n = 873) were surveyed during pregnancy and 23% (n = 378) immediately following delivery (one to three days after); 74% (n = 1208) of participants were on tenofovir‐lamivudine‐efavirenz, 13% (n = 207) on zidovudine‐lamivudine‐nevirapine, and the remaining were on less common regimens or did not know what regimen they were on. The median duration on ART was 17 months (IQR 2 to 46; range 0 to 162). Over a third (38%, n = 602) have been on ART for <6 months.

### 3.2 Proportion of participants with viral suppression or undetectable viral load

**Figure 3:**
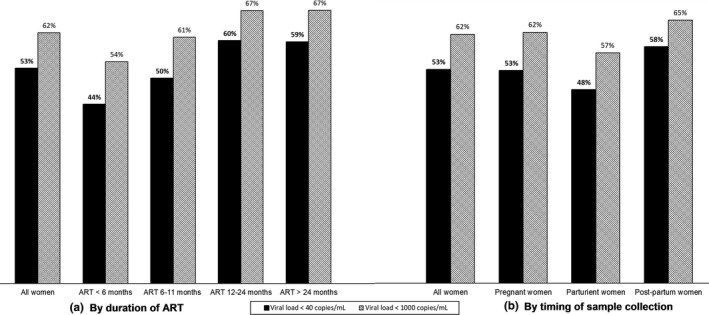
(a) Proportion of the 1623 women with viral load <1000 and <40 copies/mL by duration of ART. (b) Proportion of the 1623 women with viral load <1000 and <40 copies/mL by timing of sample collection.

**Figure 4:**
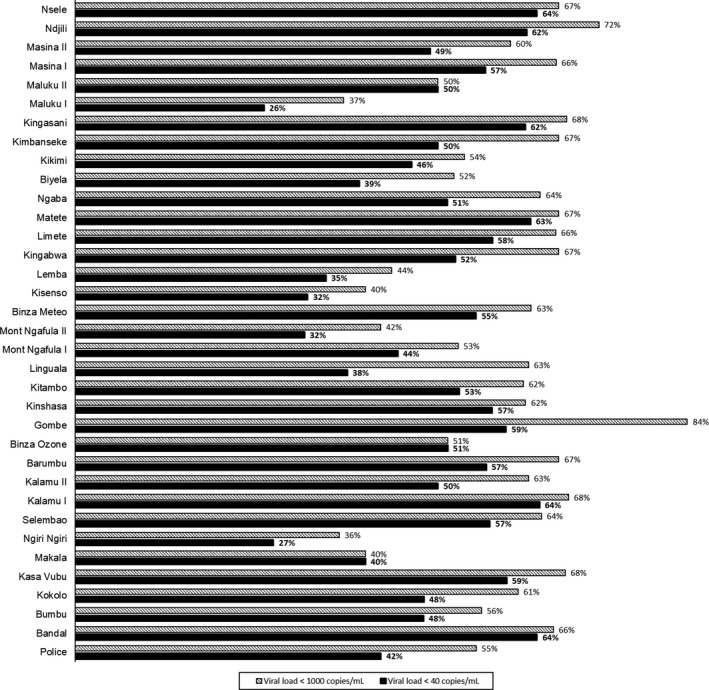
Proportion of participants with viral load <1000 and <40 copies/mL by health zone in Kinshasa.

Overall, 62% (n = 1000; 95% CI 59% to 64%) and 53% (n = 858; 95% CI 50% to 55%) of women had suppressed or undetectable viral load respectively (Figure 3). The proportion with viral suppression or undetectable viral load varied greatly by health zones, ranging from 37% in Maluku I to 84% in Gombe for suppressed viral load or from 26% in Maluku I to 64% in Bandalungwa or Kalumu I for undetectable viral load (Figure 4 and Table S3). Viral suppression and undetectable viral load also varied slightly by duration on ART with 54% (328/602) and 44%(265/602) of women on ART less than six months achieving viral suppression and undetectable viral load respectively, compared with 61%(72/119) and 50% (60/119) of women on ART between six and eleven months, 67%(118/176) and 60% (105/176) of women on ART between 12 and 24 months, and 67% (456/679) and 59% (403/679) of women on ART longer than 24 months (Figure 2).

### 3.3 Association of facility and individual characteristics with viral suppression

Table 2 summarizes the results of bivariable analysis. Overall, women receiving care in PEPFAR supported facilities were more likely to have viral load <1000 copies/mL compared to their counterparts in non‐PEPFAR supported clinics (PR 1.10; 95% CI 1.00 to 1.21). This association was strongest among breastfeeding women in postpartum period (PR 1.18; 95% CI 1.04 to 1.35). Similarly, receiving care in a hospital as opposed to health centre was statistically associated with viral suppression (PR 1.10; 95% CI 1.00 to 1.22) during pregnancy, but not at any other time. Longer duration of ART was also associated with achieving viral suppression among women enrolled during pregnancy or immediately after delivery but tended to be negatively associated with viral suppression among those enrolled in postpartum. Older or married/cohabiting women were all more likely to achieve viral suppression as were those reporting taxi/other as the primary mode of commute to the clinic as opposed to walking. Disclosure of HIV status to anyone was also positively and statistically associated with viral suppression during pregnancy and immediately after delivery, but not in the postpartum period. Similar associations were found for undetectable viral load (Table S1).

**Table 2:** Bivariable associations between facilities’, socio‐demographic and clinical characteristics and viral load <1000 copies/mL by timing of HIV viral load testing^a^

Characteristics	All women (1623)		Pregnant women (873)		Parturient women (378)		Breastfeeding mothers (378)	
	n/N (%)^b^	uPR (95% CI)^c^	n/N (%)^b^	uPR(95% CI)^c^	n/N (%)^b^	uPR(95% CI)^c^	n/N (%)^b^	uPR(95% CI)^c^
	VL <1000 copies/mL		VL <1000 copies/mL		VL <1000 copies/mL		VL <1000 copies/mL	
Location of facility attended^d^								
Peri‐urban/Rural	59/113 (52)	1	29/56 (52)	1	17/27 (63)	1	13/30 (43)	1
Urban	941/1510 (62)	1.19 (0.94, 1.51)	513/817 (63)	1.21 (0.98, 1.50)	195/345 (57)	0.90 (0.63, 1.29)	233/348 (67)	1.54 (0.90, 2.65)
PEPFAR funding of facility								
No	353/611 (58)	1	208/349 (60)	1	69/133 (52)	1	76/129 (59)	1
Yes	647/1012 (64)	1.10 (1.00, 1.21)	334/524 (64)	1.07 (0.96, 1.20)	143/239 (60)	1.14 (0.92, 1.42)	170/249 (68)	1.18 (1.04, 1.35)
Type of facility								
Health centre	418/709 (59)	1	233/399 (58)	1	75/142 (53)	1	110/168 (65)	1
Hospital	582/914 (64)	1.10 (1.00, 1.22)	309/474 (65)	1.11 (1.00, 1.24)	137/230 (60)	1.15 (0.92, 1.45)	136/210 (65)	0.99 (0.85, 1.15)
Time on ART								
<6 months	328/602 (54)	1	207/384 (54)	1	74/152 (49)	1	47/66 (71)	1
6 to 11 months	72/119 (61)	1.11 (0.93, 1.32)	26/36 (72)	1.34 (1.08, 1.67)	15/23 (65)	1.34 (0.99, 1.81)	31/60 (52)	0.73 (0.59, 0.89)
12 to 24 months	118/176 (67)	1.23 (1.07, 1.42)	54/81 (67)	1.24 (1.05, 1.46)	28/39 (72)	1.48 (1.07, 2.06)	36/56 (64)	0.90 (0.73, 1.11)
>24 months	456/679 (67)	1.23 (1.13, 1.34)	252/365 (69)	1.28 (1.17, 1.40)	86/140 (61)	1.26 (1.02, 1.56)	118/174 (68)	0.95 (0.79, 1.14)
Marital status								
Divorced/separated/widowed/never married	289/511 (57)		158/272 (58)		60/115 (52)		71/124 (57)	
Married/cohabitating	691/1078 (64)	1.14 (1.05, 1.25)	384/600 (64)	1.10 (0.99, 1.23)	145/244 (59)	1.14 (0.91, 1.43)	162/234 (69)	1.21 (1.04, 1.41)
Maternal age in years								
≤24	123/239 (51)		66/128 (52)		31/60 (52)		26/51 (51)	
25 to 34	516/848 (61)	1.18 (1.02, 1.36)	306/492 (62)	1.21 (1.00, 1.45)	87/173 (50)	0.97 (0.67, 1.39)	123/183 (67)	1.32 (1.01, 1.73)
35+	341/502 (68)	1.31 (1.14, 1.51)	170/252 (67)	1.31 (1.10, 1.57)	87/126 (69)	1.33 (0.99, 1.79)	84/124 (68)	1.33 (1.02, 1.74)
Disclosure of HIV status^e^								
No	437/787 (56)		228/426 (54)		93/193 (48)		116/168 (69)	
Yes	556/826 (67)	1.21 (1.12, 1.30)	313/446 (70)	1.31 (1.19, 1.44)	119/179 (66)	1.38 (1.13, 1.68)	124/201 (62)	0.89 (0.77, 1.04)
ART regimen								
TDF + 3TC+FEV	746/1208 (62)		410/660 (62)		174/293 (59)		162/255 (64)	
AZT + 3TC+NVP	132/207 (64)	1.04 (0.93, 1.17)	79/120 (66)	1.06 (0.91, 1.24)	13/28 (46)	0.78 (0.53, 1.13)	40/59 (68)	1.07 (0.89, 1.28)
Other	122/208 (59)	0.95 (0.84, 1.07)	53/93 (57)	0.92 (0.75, 1.12)	25/51 (49)	0.84 (0.64, 1.10)	44/64 (69)	1.08 (0.91, 1.29)
Mode of transport to the clinic								
Walking	353/601 (59)		197/332 (59)		78/140 (56)		78/129 (60)	
Taxi/other	627/988 (63)	1.07 (0.98, 1.16)	345/541 (64)	1.08 (0.96, 1.21)	127/218 (58)	1.04 (0.85, 1.27)	78/129 (60)	1.13 (0.95, 1.35)
Primigravida								
Yes	86/145 (59)		50/86 (58)		18/36 (50)		18/23 (78)	
No	894/1445 (62)	1.04 (0.90, 1.21)	492/787 (63)	1.08 (0.88, 1.31)	187/323 (58)	1.16 (0.85, 1.57)	215/335 (64)	0.82 (0.66, 1.01)
Alcohol consumption								
>4 times/month	60/95 (63)		31/55 (56)		17/26 (65)		12/14 (86)	
1 to 3 times/month	216/357 (61)		131/207 (63)		44/81 (54)		41/69 (59)	
No	704/1137 (62)	1.02 (0.95, 1.10)	380/610 (62)	1.02 (0.95, 1.10)	144/252 (57)	1.01 (0.91, 1.11)	180/275 (66)	1.01 (0.84, 1.22)
Educational level								
Primary	136/221 (62)		70/111 (63)		30/55 (55)		36/55 (65)	
Secondary	686/1118 (61)	1.00 (0.88, 1.13)	374/601 (62)	0.99 (0.84, 1.16)	146/254 (57)	1.06 (0.84, 1.32)	166/263 (63)	0.96 (0.78, 1.19)
Tertiary	157/249 (63)	1.02 (0.88, 1.19)	98/160 (61)	0.97 (0.81, 1.17)	28/49 (57)	1.06 (0.77, 1.46)	31/40 (78)	1.18 (0.92, 1.52)
SES in quintile^f^								
0 (Lowest)	175/295 (59)		90/155 (58)		33/60 (55)		52/80 (65)	
1	168/274 (61)	1.04 (0.92, 1.18)	89/147 (61)	1.04 (0.87, 1.25)	36/62 (58)	1.07 (0.75, 1.54)	43/65 (66)	1.02 (0.84, 1.23)
2	186/289 (64)	1.08 (0.94, 1.25)	114/173 (66)	1.13 (0.95, 1.35)	41/70 (59)	1.08 (0.75, 1.55)	31/46 (67)	1.03 (0.82, 1.31)
3	161/271 (59)	1.00 (0.85, 1.18)	112/181 (62)	1.07 (0.90, 1.26)	23/46 (50)	0.93 (0.57, 1.52)	26/44 (59)	0.90 (0.67, 1.21)
4 (Highest)	193/297 (65)	1.10 (0.97, 1.24)	126/198 (64)	1.10 (0.93, 1.29)	37/53 (70)	1.30 (0.98, 1.72)	30/46 (65)	1.00 (0.77, 1.30)

ART, antiretroviral therapy; CI, confidence interval; SES, socio‐economic status; uPR, unadjusted prevalence ratio; VL, viral load.

^a^The analytical sample was derived from the enrolment data of an ongoing cluster randomized controlled trial, aimed at evaluating the effect of data‐driven continuous quality improvement on long‐term ART outcomes in Kinshasa, Democratic Republic of Congo; ^b^frequencies might not add up to the total for the category, because of missing data. Percentages are for rows; ^c^estimated by log binomial models, where general estimating equation was used to adjust for within health zone clustering; ^d^facility at which participants were enrolled; ^e^self‐reported disclosure of HIV status to anyone; ^f^calculated using principal component analysis and categorized in five quintile groups.

In a multivariable analysis that included the location and type of facility, source of international funding/support, duration of ART, age, disclosure of HIV status, marital status, and mode of transport to clinic, the results did not change substantially (Table 3). Among breastfeeding women, receiving care in a PEPFAR supported clinic remained statistically associated with viral suppression: aPR 1.17; 95% CI 1.02, 1.34 respectively. Disclosure of HIV status remained associated with viral suppression among pregnant women and those surveyed immediately after delivery: aPR 1.23; 95% CI 1.11, 1.36 and 1.39; 95% CI 1.16, 1.66 respectively. Despite not statistically significant, disclosure of HIV status and not walking to the clinic were both negatively associated with viral suppression among breastfeeding women (aPR 0.88; 95% CI 0.75, 1.02). Using <40 copies/mL as cut‐off did not change the results substantially (Table S2).

**Table 3:** Multivariable associations between facility's, socio‐demographic and clinical characteristics and viral load <1000 copies/mL, by timing of viral load testing^a^

Baseline covariates	All women (1623)	Pregnant women (873)	Parturient women (378)	Breastfeeding mothers (378)
	APR^b^ (95% CI)	APR^b^ (95% CI)	APR^b^ (95% CI)	aPR^b^ (95% CI)
Location of facility^c^ attended				
Peri‐urban/rural	1	1	1	1
Urban	1.24 (1.00, 1.54)	1.26 (1.00, 1.59)	1.04 (0.78, 1.39)	1.50 (0.86, 2.59)
Type of facility				
Health centre	1	1	1	1
Hospital	1.05 (0.97, 1.15)	1.09 (1.00, 1.19)	1.16 (0.96, 1.41)	0.97 (0.87, 1.08)
PEPFAR funding of facility				
No	1	1	1	1
Yes	1.09 (1.00, 1.19)	1.07 (0.96, 1.18)	1.14 (0.92, 1.41)	1.17 (1.02, 1.34)
Time on ART				
<6 months	1	1	1	1
6 to 11 months	1.07 (0.91, 1.27)	1.28 (1.05, 1.56)	1.19 (0.81, 1.75)	0.73 (0.59, 0.90)
12 to 24 months	1.19 (1.03, 1.37)	1.15 (0.97, 1.36)	1.42 (1.12, 1.81)	0.90 (0.70, 1.15)
>24 months	1.14 (1.02, 1.27)	1.18 (1.04, 1.33)	1.05 (0.86, 1.27)	0.94 (0.79, 1.11)
Maternal age in years				
≤24	1	1	1	1
25 to 34	1.11 (0.96, 1.28)	1.12 (0.92, 1.35)	0.86 (0.60, 1.23)	1.22 (0.94, 1.58)
35+	1.21 (1.05, 1.39)	1.17 (0.97, 1.41)	1.17 (0.85, 1.66)	1.28 (0.98, 1.66)
Disclosure of HIV status^d^				
No	1	1	1	1
Yes	1.15 (1.07, 1.25)	1.23 (1.11, 1.36)	1.39 (1.16, 1.66)	0.88 (0.75, 1.02)
Marital status				
Divorced/separated/widowed/never married	1	1	1	1
Married/cohabitating	1.09 (1.00, 1.19)	1.06 (0.95, 1.18)	1.12 (0.90, 1.39)	1.12 (0.98, 1.29)
Mode of transport to the clinic				
Walking	1	1		
Taxi/other	1.00 (0.91, 1.08)	0.98 (0.87, 1.10)	0.89 (0.73, 1.07)	1.08 (0.91, 1.30)

aPR, adjusted prevalence ratio; ART, antiretroviral therapy; VL, viral load.

^a^The analytical sample was derived from the enrolment data of an ongoing cluster randomized controlled trial, aimed at evaluating the effect of data‐driven continuous quality improvement on long‐term ART outcomes in Kinshasa, Democratic Republic of Congo. We retained participants that had available data on HIV viral load testing; ^b^estimated by log binomial models, adjusted for all covariates in the table, and where general estimating equation was used to adjust for within health zone clustering; ^c^facility at which participants attend PMTCT visits; ^d^self‐reported disclosure of HIV status to anyone.

## 4 Discussion

To the best of our knowledge, this is the first study that aimed at estimating viral suppression at the population level among pregnant and breastfeeding women in routine HIV care. Our results show that, in the city‐province of Kinshasa in DRC, only 62% and 53% of pregnant and breastfeeding women living with HIV and in care, had viral load <1000 and <40 copies/mL respectively. Even among women who had been on ART longer than 12 months, the proportion with viral load <1000 or <40 copies/mL only increased to 67% or 60% respectively. Given the known tendency of DBS compared to plasma samples to systematically underestimate viral load 18, the true proportion of women with suppressed viral load is probably even lower.

The *Start Free, Stay Free, AIDS Free* ambitious target of reducing new paediatric HIV infection to 20,000 by 2020 is based on the premise that at least 95% of PWLHIV who are on ART will achieve and sustain suppressed viral load during pregnancy, delivery and throughout the breastfeeding period 2. Data on viral load among pregnant and breastfeeding women in sub‐Saharan Africa are limited and mostly come from study cohorts, principally from South Africa. In a cohort of newly diagnosed pregnant women, enrolled in the control arm of an intervention study before 28 weeks of gestation in some of the same clinics participating in the current study, 70% had undetectable viral load at six weeks postpartum although only 78% were on ART at enrolment 7. Similar levels of undetectable viral load were observed at delivery in a cohort of pregnant women initiated on ART at a median of 20 weeks of gestation in South Africa, 10 and in Malawi 19, 20. These data from cohort studies, combined with high levels of viral suppression in centrally located health zones suggest that with increased attention to the quality of care of the kind that is associated with cohort studies (avoidance of stockouts, better training and supervision of providers among others), higher rates of viral suppression can be achieved among pregnant and breastfeeding women in care, no matter the settings. However, even in Southern Africa were over 93% of PWLHIV were estimated to be receiving ART in 2017, estimates of mother‐to‐child transmission still stand around 10% 21, suggesting similar difficulties as in Kinshasa to achieve and sustained high level of undetectable viral load in routine care settings.

The issue of high loss‐to‐follow‐up immediately after ART initiation has been at the frontline of PMTCT research, since the start of the option B+ era 4, 15, 22, 23, 24. This would implicate that women who are at the highest risk for non‐adherence tend to drop‐out the soonest. Overall, 46% of women in our study had been on ART for <12 months. The proportion of women with viral <1000 or < 40 copies/mL increased from 54% and 44% respectively, among participants who had been on ART for <6 months, to 67% and 60% among those who had been on ART for 12 to 24 months or >24 months and the differences were statistically significant. Similar differences were observed among women tested during pregnancy or in the postpartum period, suggesting the low level of viral suppression is mainly attributable to poor adherence, even in those who have been retained in care for quite some time.

Over 74% of participants were on TDF/3TC/EFV, a newer regimen that was introduced in this population along with the 2013 WHO recommendation. Yet, the prevalence of detectable viral load or viral load ≥1000 copies/mL did not differ from women on older regimens, mainly AZT/3TC/NVP. Although, we do not have a second viral load to confirm virologic failure 13, it is particularly concerning to see that a third of participants who had been on ART for ≥12 months had viral load above 1000 copies/mL. In a recent survey of 1064 Cameroonian adults, drug resistance was observed in 63% and 88% of participants with viral load ≥1000 copies/mL and who were on ART between 12 to 24 months and 48 to 60 months respectively 25. If the same level of drug resistance were to be true in our sample, more than 20% of pregnant and breastfeeding women in care in Kinshasa would have drug resistance to potentially TDF and EFV. These findings support the call from a recent consensus statement on research priorities to inform “treat all” implementation in sub‐Saharan Africa for research to estimate the incidence and prevalence of drug resistance 26.

The usual clinic and socio‐demographic characteristics known to be associated with retention in care and adherence were also associated with higher viral suppression in our population. At the individual level, being married or cohabiting, not walking to the clinic, disclosure of HIV status were all positively associated with viral suppression 27, 28, 29. Nonetheless, our finding in that disclosure of HIV status to anyone might negatively affect viral suppression in breastfeeding women warrants further investigations. Emerging data from South Africa suggest that disclosure is not always universally positive and may depend on the person to whom the status is disclosed to 30, 31.

At the facility level, receiving care in a hospital, in an urban area, or in a PEPFAR‐supported facility were all positively associated with viral suppression, particularly at delivery and in the postpartum period, reflecting the possible higher degree of proficiency among clinics that receive technical support from PEPFAR implementing partners 32, 33. However, selection of clinics supported by PEPFAR was not done randomly and because of the cross‐sectional design of the study, it is not possible to ascertain whether this association is a result of PEPFAR selecting clinics that are known to have the capacity to deliver better HIV care or the result of additional technical support to these clinics. Stockouts of HIV commodities including ART are common in health facilities across Kinshasa even when such commodities are available at local upstream warehouses 34. It is possible that the additional support from PEPFAR, through regular supervision is effective in reducing the impact of such stockouts on ART adherence.

Selection of clinics was based on their size as opposed to probability sampling, meaning that our sample may not be representative of all PWLHIV in the province. However, because all 35 health zones in the Kinshasa province were represented and virtually all women receiving ART care in these clinics were enrolled, our results apply to the vast majority of women in care in the province. The study has additional limitations. First, because of the cross‐sectional nature of the design, we do not have a second sample from participants to confirm viral failure among those with viral load ≥1000 copies/mL. Similarly, drug resistance testing was not planned in the study and is not routinely available in Kinshasa. Confirming viral failure and estimating the level of drug resistance in this population is urgently needed to inform choice of treatment regimen for both pregnant and breastfeeding women in DRC and their HIV‐positive infants. In addition, because of the cross‐sectional design we were also unable to account for selective drop‐out of potentially non‐adherent women. Second, few eligible participants either did not agree to provide a blood sample for viral load testing, or the sample was not properly collected or handled. However, given that virtually none of the women knew their viral load at the time, it is unlikely that inclusion in this analysis was related to actual value of viral load. Third, we did not collect data on drug stockouts during the study. It is possible that many of the women with unsuppressed viral load were simply not taking any medication at the time of the blood collection 34. Fourth, as mentioned earlier, DBS viral load systematically underestimates plasma viral load 18, suggesting that the true proportion of women with viral load ≥1000 copies/mL is likely underestimated.

## 5 Conclusions

Close to half of pregnant and breastfeeding women receiving ART care in MCH clinics in Kinshasa, had detectable viral load. Even among women who had been on ART for longer than 12 months, at least a third had viral load ≥1000 copies/mL suggesting a potential high level of drug resistance that needs to be urgently investigated. The observed high level of detectable viral load suggests that high ART coverage alone without substantial efforts to improve the quality of care for pregnant and breastfeeding women, will not be enough to achieve the goal of virtual elimination of vertical HIV transmission in high‐burden and limited resources settings like DRC.

## Competing interests

No author had a competing interest to declare.

## Authors’ contributions

MY, FB and EO conceptualized the study and acquired funding. CM, MT, NLRR, FM, BK, EO and MY acquired the data. CM and MY performed data analysis and drafted the first manuscript version. All authors read and approved the final manuscript.

## Supporting information


**Table S1.** Bivariable associations between facilities’, socio‐demographic and clinical characteristics and viral load <40 copies/mL by timing of HIV viral load testing^a^
**Table S2.** Multivariable associations between facility characteristics, socio‐demographic and clinical characteristics of 1623 women tested for HIV viral load in 105 clinics in Kinshasa between November 2016 and July 2018 and viral load <40 copies/mL, stratified by timing of viral load testing^a^
**Table S3.** Prevalence of VL<1000 copies/mL and VL<40 cp/mL by health zone in Kinshasa. Democratic Republic of Congo
