# Supplementary material for: HIV viral suppression among pregnant and breastfeeding women in routine care in the Kinshasa province: a baseline evaluation of participants in CQI‐PMTCT study
Source: J Int AIDS Soc. 2019 Sep 8;22(9):e25376. doi: 10.1002/jia2.25376 (PMC6732557; doi:10.1002/jia2.25376)
Supplement: Supplementary file 1 — Table S1. Bivariable associations between facilities’, socio‐demographic and clinical characteristics and viral load <40 copies/mL by timing of HIV viral load testinga Table S2. Multivariable associations between facility characteristics, socio‐demographic and clinical characteristics of 1623 women tested for HIV viral load in 105 clinics in Kinshasa between November 2016 and July 2018 and viral load <40 copies/mL, stratified by timing of viral load testinga Table S3. Prevalence of VL<1000 copies/mL and VL<40 cp/mL by health zone in Kinshasa. Democratic Republic of Congo [file JIA2-22-e25376-s001.docx]

Supplemental Tables

**Table S1. Bivariable associations between facilities', socio-demographic and clinical characteristics and viral load < 40 cp/mL by timing of HIV viral load testing ^a^**

| **Characteristics** | **All women (1623)** | |  | **Pregnant women (873)** | |  | **Parturient women (378)** | |  | **Breastfeeding mothers (378)** | |
| --- | --- | --- | --- | --- | --- | --- | --- | --- | --- | --- | --- |
|  | n/N(%) ^b^ | uPR(95% CI) ^c^ |  | n/N(%) ^b^ | uPR(95% CI) ^c^ |  | n/N(%) ^b^ | uPR(95% CI) ^c^ |  | n/N(%) ^b^ | uPR(95% CI) ^c^ |
|  | VL<40 cp/mL |  |  | VL<40 cp/mL |  |  | VL<40 cp/mL |  |  | VL<40 cp/mL |  |
| **Location of facility attended** |  |  |  |  |  |  |  |  |  |  |  |
| Peri-urban/Rural | 51/113(45) | 1 |  | 25/56(45) | 1 |  | 14/27(52) | 1 |  | 12/30(40) | 1 |
| Urban | 807/1510(53) | 1.18(0.85,1.62) |  | 434/817(53) | 1.20(0.90,1.60) |  | 164/345(48) | 0.93(0.54,1.58) |  | 209/348(60) | 1.51(0.86,2.63) |
| **PEPFAR funding of facility ^d^** |  |  |  |  |  |  |  |  |  |  |  |
| No | 295/611(48) | 1 |  | 175/349(50) | 1 |  | 57/133(43) | 1 |  | 63/129(49) | 1 |
| Yes | 563/1012(56) | 1.14(1.02,1.27) |  | 284/524(54) | 1.09(0.96,1.24) |  | 121/239(51) | 1.18(0.93,1.50) |  | 158/249(63) | 1.35(1.18,1.55) |
| **Type of facility** |  |  |  |  |  |  |  |  |  |  |  |
| Health center | 358/709(50) | 1 |  | 200/399(50) | 1 |  | 57/142(40) | 1 |  | 101/168(60) | 1 |
| Hospital | 500/914(55) | 1.10(0.98,1.23) |  | 259/474(55) | 1.09(0.96,1.25) |  | 121/230(53) | 1.31(1.04,1.65) |  | 120/210(57) | 0.95(0.80,1.13) |
| **Time on ART** |  |  |  |  |  |  |  |  |  |  |  |
| < 6 months | 265/602(44) | 1 |  | 166/384(43) | 1 |  | 58/152(38) | 1 |  | 41/66(62) | 1 |
| 6-11 months | 60/119(50) | 1.13(0.88,1.46) |  | 22/36(61) | 1.42(1.10,1.84) |  | 12/23(52) | 1.36(0.78,2.36) |  | 26/60(43) | 0.70(0.50,0.96) |
| 12-24 months | 105/176(60) | 1.36(1.17,1.58) |  | 46/81(57) | 1.32(1.05,1.66) |  | 26/39(67) | 1.76(1.30,2.39) |  | 33/56(59) | 0.95(0.71,1.27) |
| > 24 months | 403/679(59) | 1.34(1.20,1.49) |  | 222/365(61) | 1.41(1.22,1.63) |  | 74/140(53) | 1.38(1.12,1.71) |  | 107/174(61) | 0.99(0.79,1.23) |
| **Marital status** |  |  |  |  |  |  |  |  |  |  |  |
| Divorced/separated/ widowed/never married | 241/511(47) | 1 |  | 127/272(47) | 1 |  | 52/115(45) | 1 |  | 62/124(50) | 1 |
| Married/cohabitating | 597/1078(55) | 1.19(1.07,1.32) |  | 332/600(55) | 1.19(1.02,1.39) |  | 119/244(49) | 1.08(0.82,1.43) |  | 146/234(62) | 1.25(1.04,1.49) |
| **Age (median [IQR])** |  |  |  |  |  |  |  |  |  |  |  |
| ≤ 24 | 100/239(42) | 1 |  | 52/128(41) | 1 |  | 24/60(40) | 1 |  | 24/51(47) | 1 |
| 25-34 | 444/848(52) | 1.25(1.07,1.45) |  | 259/492(53) | 1.30(1.04,1.61) |  | 74/173(43) | 1.07(0.74,1.54) |  | 111/183(61) | 1.29(0.93,1.79) |
| 35+ | 294/502(59) | 1.39(1.18,1.64) |  | 148/252(59) | 1.45(1.15,1.82) |  | 73/126(58) | 1.45(1.04,2.02) |  | 73/124(59) | 1.25(0.91,1.71) |
| **Disclosure of HIV status** |  |  |  |  |  |  |  |  |  |  |  |
| No | 369/787(47) | 1 |  | 186/426(44) | 1 |  | 79/193(41) | 1 |  | 104/168(62) | 1 |
| Yes | 482/826(58) | 1.24(1.13,1.37) |  | 272/446(61) | 1.40(1.23,1.59) |  | 99/179(55) | 1.35(1.08,1.69) |  | 111/201(55) | 0.89(0.74,1.07) |
| **ART regimen** |  |  |  |  |  |  |  |  |  |  |  |
| TDF+3TC+FEV | 628/1208(52) | 1 |  | 341/660(52) | 1 |  | 143/293(49) | 1 |  | 144/255(57) | 1 |
| AZT+3TC+NVP | 118/207(57) | 1.11(0.97,1.27) |  | 71/120(59) | 1.15(0.95,1.38) |  | 12/28(43) | 0.86(0.58,1.30) |  | 35/59(59) | 1.05(0.85,1.30) |
| Other | 112/208(54) | 1.04(0.91,1.17) |  | 47/93(51) | 0.98(0.79,1.21) |  | 23/51(45) | 0.95(0.67,1.33) |  | 42/64(66) | 1.16(0.96,1.40) |
| **Mode of transport to the clinic** |  |  |  |  |  |  |  |  |  |  |  |
| Walking | 301/601(50) | 1 |  | 166/332(50) | 1 |  | 65/140(46) | 1 |  | 70/129(54) | 1 |
| Taxi/other | 537/988(54) | 1.06(0.96,1.16) |  | 293/541(54) | 1.09(0.96,1.24) |  | 106/218(49) | 1.04(0.82,1.32) |  | 138/229(60) | 1.11(0.90,1.37) |
| **Primigravida** |  |  |  |  |  |  |  |  |  |  |  |
| yes | 72/145(50) | 1 |  | 40/86(47) | 1 |  | 15/36(42) | 1 |  | 17/23(74) | 1 |
| no | 766/1445(53) | 1.06(0.89,1.28) |  | 419/787(53) | 1.15(0.89,1.48) |  | 156/323(48) | 1.16(0.86,1.57) |  | 191/335(57) | 0.77(0.58,1.00) |
| **Educational level** |  |  |  |  |  |  |  |  |  |  |  |
| Primary | 121/221(55) | 1 |  | 63/111(57) | 1 |  | 24/55(44) | 1 |  | 34/55(62) | 1 |
| Secondary | 588/1118(53) | 0.96(0.84,1.10) |  | 314/601(52) | 0.92(0.77,1.11) |  | 121/254(48) | 1.09(0.81,1.48) |  | 153/263(58) | 0.94(0.72,1.22) |
| Tertiary | 128/249(51) | 0.94(0.80,1.12) |  | 82/160(51) | 0.90(0.74,1.11) |  | 25/49(51) | 1.18(0.79,1.78) |  | 21/40(53) | 0.85(0.58,1.25) |
| **SES in quintile ^f^** |  |  |  |  |  |  |  |  |  |  |  |
| 0 (Lowest) | 163/295(55) | 1 |  | 81/155(52) | 1 |  | 32/60(53) | 1 |  | 50/80(63) | 1 |
| 1 | 141/274(51) | 0.94(0.80,1.09) |  | 75/147(51) | 0.98(0.77,1.24) |  | 27/62(44) | 0.85(0.57,1.27) |  | 39/65(60) | 0.96(0.77,1.21) |
| 2 | 154/289(53) | 0.96(0.83,1.11) |  | 95/173(55) | 1.05(0.87,1.27) |  | 33/70(47) | 0.91(0.64,1.29) |  | 26/46(57) | 0.90(0.70,1.17) |
| 3 | 134/271(49) | 0.89(0.74,1.06) |  | 93/181(51) | 0.98(0.81,1.19) |  | 18/46(39) | 0.74(0.38,1.46) |  | 23/44(52) | 0.84(0.59,1.19) |
| 4 (Highest) | 161/297(54) | 0.98(0.85,1.13) |  | 107/198(54) | 1.03(0.85,1.25) |  | 32/53(60) | 1.20(0.86,1.68) |  | 22/46(48) | 0.77(0.53,1.10) |

^a^ The analytical sample was derived from the enrollment data of an ongoing cluster randomized controlled trial, aimed at evaluating the effect of data-driven continuous quality improvement on long-term ART outcomes in Kinshasa, Democratic Republic of Congo. We retained participants that had available data on HIV viral load testing. ^b^ Frequencies might not add up to the total for the category, because of missing data. Percentages are for rows. ^c^ Estimated by log binomial models, where general estimating equation was used to adjust for within health facilities clustering. ^d^ Facility at which participants attended PMTCT visits. #Self-reported disclosure of HIV status to anyone. ^f^ Calculated using principal component analysis and categorized in five quintile groups. Abbreviations: ART, Antiretroviral therapy; uPR, Unadjusted prevalence ratio; CI, Confidence interval; VL, Viral load; SES, Socio-economic status.

| **Baseline covariates** | **All women (1623)** | |  | **Pregnant women (873)** | |  | **Parturient women (378)** | |  | **Breastfeeding mothers (378)** | |  |
| --- | --- | --- | --- | --- | --- | --- | --- | --- | --- | --- | --- | --- |
|  | aPR ^b^ | 95% CI |  | aPR ^b^ | 95% CI |  | aPR ^b^ | 95% CI |  | aPR ^b^ | 95% CI |  |
| **Location of facility attended ^c^** |  |  |  |  |  |  |  |  |  |  |  |  |
| Peri-urban/rural | 1 |  |  | 1 |  |  | 1 |  |  | 1 |  |  |
| Urban | 1.25 | (0.94,1.66) |  | 1.26 | (0.97,1.65) |  | 1.08 | (0.72,1.62) |  | 1.53 | (0.86,2.72) |  |
|  |  |  |  |  |  |  |  |  |  |  |  |  |
| **Type of facility** |  |  |  |  |  |  |  |  |  |  |  |  |
| Health center |  |  |  |  |  |  |  |  |  |  |  |  |
| Hospital | 1.04 | (0.95,1.14) |  | 1.05 | (0.95,1.15) |  | 1.33 | (1.08,1.63) |  | 0.95 | (0.84,1.06) |  |
|  |  |  |  |  |  |  |  |  |  |  |  |  |
| **PEPFAR funding of facility** |  |  |  |  |  |  |  |  |  |  |  |  |
| No | 1 |  |  | 1 |  |  | 1 |  |  | 1 |  |  |
| Yes | 1.13 | (1.02,1.25) |  | 1.10 | (0.98,1.22) |  | 1.21 | (0.96,1.53) |  | 1.36 | (1.16,1.60) |  |
|  |  |  |  |  |  |  |  |  |  |  |  |  |
| **Time on ART** |  |  |  |  |  |  |  |  |  |  |  |  |
| < 6 months | 1 |  |  | 1 |  |  | 1 |  |  | 1 |  |  |
| 6-11 months | 1.11 | (0.89,1.38) |  | 1.35 | (1.07,1.71) |  | 1.21 | (0.70,2.10) |  | 0.74 | (0.57,0.97) |  |
| 12-24 months | 1.30 | (1.12,1.51) |  | 1.21 | (0.98,1.50) |  | 1.67 | (1.26,2.22) |  | 0.98 | (0.74,1.29) |  |
| > 24 months | 1.24 | (1.10,1.39) |  | 1.27 | (1.09,1.48) |  | 1.14 | (0.93,1.40) |  | 1.02 | (0.83,1.26) |  |
|  |  |  |  |  |  |  |  |  |  |  |  |  |
| **Age** |  |  |  |  |  |  |  |  |  |  |  |  |
| ≤ 24 | 1 |  |  | 1 |  |  | 1 |  |  | 1 |  |  |
| 25-34 | 1.15 | (0.99,1.34) |  | 1.17 | (0.95,1.46) |  | 0.94 | (0.65,1.35) |  | 1.15 | (0.86,1.54) |  |
| 35+ | 1.25 | (1.06,1.47) |  | 1.26 | (1.00,1.59) |  | 1.25 | (0.89,1.74) |  | 1.18 | (0.89,1.57) |  |
|  |  |  |  |  |  |  |  |  |  |  |  |  |
| **Disclosure of HIV status ^d^** |  |  |  |  |  |  |  |  |  |  |  |  |
| No | 1 |  |  |  |  |  |  |  |  |  |  |  |
| Yes | 1.16 | (1.05,1.29) |  | 1.27 | (1.10,1.46) |  | 1.35 | (1.10,1.65) |  | 0.89 | (0.75,1.07) |  |
|  |  |  |  |  |  |  |  |  |  |  |  |  |
| **Marital status** |  |  |  |  |  |  |  |  |  |  |  |  |
| Divorced/separated/ widowed/never married | 1 |  |  | 1 |  |  | 1 |  |  | 1 |  |  |
| Married/cohabitating | 1.12 | (1.01,1.24) |  | 1.12 | (0.96,1.30) |  | 1.03 | (0.79,1.35) |  | 1.10 | (0.94,1.27) |  |
|  |  |  |  |  |  |  |  |  |  |  |  |  |
| **Mode of transport to the clinic** |  |  |  |  |  |  |  |  |  |  |  |  |
| Walking | 1 |  |  | 1 |  |  | 1 |  |  | 1 |  |  |
| Taxi/other | 0.98 | (0.89,1.08) |  | 0.98 | (0.87,1.11) |  | 0.85 | (0.69,1.06) |  | 1.05 | (0.85,1.30) |  |

**Table S2. Multivariable associations between facility characteristics, socio-demographic and clinical characteristics of 1623 women tested for HIV viral load in 105 clinics in Kinshasa between November 2016 and July 2018 and viral load <40 copies/mL, stratified by timing of viral load testing ^a^**

^a^ The analytical sample was derived from the enrollment data of an ongoing cluster randomized controlled trial, aimed at evaluating the effect of data-driven continuous quality improvement on long-term ART outcomes in Kinshasa, Democratic Republic of Congo. We retained participants that had available data on HIV viral load testing. ^b^ Estimated by log binomial models, adjusted for all covariates in the table, and where general estimating equation was used to adjust for within health facilities clustering. ^c^ Facility at which participants attend PMTCT visits. ^d^ Self-reported disclosure of HIV status to anyone. Abbreviations: ART, Antiretroviral therapy; aPR, Adjusted prevalence ratio; VL, Viral load

**Table S3. Prevalence of VL<1000 copies/mL and VL<40 cp/mL by health zone in Kinshasa. Democratic Republic of Congo**

| **Health zones** | VL<1000 cp/mL |  | VL<40 cp/mL |
| --- | --- | --- | --- |
|  | n/N(%) ^b^ |  | n/N(%) ^b^ |
| Police | 21/38(55) |  | 16/38(42) |
| Bandalungwa | 29/44(66) |  | 28/44(64) |
| Bumbu | 42/75(56) |  | 36/75(48) |
| Kokolo | 47/77(61) |  | 37/77(48) |
| Kasa Vubu | 25/37(68) |  | 22/37(59) |
| Makala | 10/25(40) |  | 10/25(40) |
| Ngiri Ngiri | 4/11(36) |  | 3/11(27) |
| Selembao | 9/14(64) |  | 8/14(57) |
| Kalamu I | 17/25(68) |  | 16/25(64) |
| Kalamu II | 10/16(63) |  | 8/16(50) |
| Barumbu | 20/30(67) |  | 17/30(57) |
| Binza Ozone | 19/37(51) |  | 19/37(51) |
| Gombe | 27/32(84) |  | 19/32(59) |
| Kinshasa | 54/87(62) |  | 50/87(57) |
| Kitambo | 42/68(62) |  | 36/68(53) |
| Linguala | 15/24(63) |  | 9/24(38) |
| Mont Ngafula I | 19/36(53) |  | 16/36(44) |
| Mont Ngafula II | 8/19(42) |  | 6/19(32) |
| Binza Meteo | 66/105(63) |  | 58/105(55) |
| Kisenso | 10/25(40) |  | 8/25(32) |
| Lemba | 24/55(44) |  | 19/55(35) |
| Kingabwa | 28/42(67) |  | 22/42(52) |
| Limete | 53/80(66) |  | 46/80(58) |
| Matete | 34/51(67) |  | 32/51(63) |
| Ngaba | 25/39(64) |  | 20/39(51) |
| Biyela | 12/23(52) |  | 9/23(39) |
| Kikimi | 22/41(54) |  | 19/41(46) |
| Kimbanseke | 16/24(67) |  | 12/24(50) |
| Kingasani | 107/158(68) |  | 98/158(62) |
| Maluku I | 7/19(37) |  | 5/19(26) |
| Maluku II | 3/6(50) |  | 3/6(50) |
| Masina I | 61/92(66) |  | 52/92(57) |
| Masina II | 27/45(60) |  | 22/45(49) |
| Ndjili | 65/90(72) |  | 56/90(62) |
| Nsele | 22/33(67) |  | 21/33(64) |
